# Thirty years after the Cairo declaration on population & development: have family planning measurements caught up?

**DOI:** 10.3389/fgwh.2025.1548447

**Published:** 2025-06-13

**Authors:** Nour Horanieh, Alice Witt, Marieme Fall, Eloisa Montt-Maray, Lamiah Adamjee, Elizabeth Larson, Thais Gonzalez-Capella, Beniamino Cislaghi

**Affiliations:** ^1^ Department of Global Health and Development, London School of Hygiene and Tropical Medicine, London, United Kingdom; ^2^ Department of Family and Community Medicine, College of Medicine, King Saud University, Riyadh, Saudi Arabia; ^3^ Department of Epidemiology, Biostatistics and Occupational Health, McGill University, Montreal, QC, Canada; ^4^ Center on Gender Equity and Health, University of California San Diego, La Jolla, CA, United States; ^5^ School of Social Sciences, Makerere University, Kampala, Uganda; ^6^ Institute for Development Studies, Brighton, United Kingdom

**Keywords:** family planning, ethics, measurements, indicators, autonomy

## Abstract

**Introduction:**

The 1994 International Conference on Population Development (ICPD) initiated the transition of family planning (FP) programmes from focusing on population control to promoting human rights and women's empowerment. The indicators used to measure success of FP programmes, however, continue to focus on estimating modern contraceptive uptake. Contraceptive Prevalence Rate (CPR) and unmet need are the main indicators used. We aim to assess the views of those working within the FP community from the Global North and South on the use of current indicators for FP programmes. While there have been calls for new measures, understanding the barriers to changing existing ones is essential for adopting and implanting these new measures.

**Methods:**

We conducted semi-structured interviews with 31 participants from five distinct groups; academics, NGO workers, government officials, funding agency workers and advocates. Participants were working in countries worldwide, including both the Global North and South; the latter were mostly based in Francophone West Africa. Interviews explored several themes including FP targets and indicators. We applied a thematic analysis.

**Results:**

Participants' views ranged from those who believed in the need to eradicate current indicators from FP programmes to those expressing contentment with current indicators and their benefit in measuring success. Most of the participants acknowledged the benefit of indicators in assessing progress or as a starting point, yet they identified multiple limitations to their use, including the possibility of implicit coercion, skewing training to focus on long-acting reversible contraceptives (LARCs) promotion, and prioritising modern contraceptive methods over natural ones. Some expressed anxiety that challenging the status quo could lead to funding cuts. Participants identified challenges in adopting new indicators and emphasised that funding for FP programs remains largely concentrated among international agencies based in the Global North, which results in maintaining certain traditional demographic approaches.

**Conclusion:**

Current indicators affect the understanding of success of FP programmes and influence how FP services translate on the ground. We provide international stakeholders' perspectives on the barriers to be overcome to support development of new indicators, including non-use of contraception as a success as long as it is a full, free and informed choice.

## 1 Introduction

Historically, family planning (FP) programmes have been conceived as a solution to curb rapid population growth, promoting socio-economic development, improving maternal and child health outcomes, and more recently ensuring environmental sustainability (1, 2). After World War II, concerns over population growth and stagnant agricultural production fuelled neo-Malthusian perspectives in Western Europe, leading to a push for “population control” programmes. This was a contrasting perspective from the previous push for women's choice and autonomy, which had gained momentum in the 1950s with the advent of the oral contraceptive pill (3). In the 1970s, feminist and social activists, as well as academics, critiqued population control initiatives for prioritizing economic outcomes over individual reproductive rights, particularly as some countries adopted coercive practices like China's One-Child Policy and forced sterilizations in Peru and India (4–8).

Most public health interventions are assessed by their success in preventing morbidity and mortality. However, since the main objective of FP programmes is preventing unwanted pregnancy, they require their own measures of success. Historically and today, the most common programme indicators are total fertility rate (TFR), unmet need, and modern contraceptive prevalence rate (mCPR) (9, 10). These indicators were designed in the 1960s to capture data at the population rather than the individual level, and few changes have been made to update them to ensure individual choice is captured (11, 12). For example, the unmet need indicator was developed in the 1970s by demographers to identify fertility patterns and contraceptive use, especially in low- and middle-income countries (LMICs). The indicator identifies populations not using contraceptive methods to be targeted by interventions like demand generation strategies that include community-based interventions, financial incentives, and media campaigns (10, 13). Though “unmet need” is defined as the percentage of fecund and sexually active women who do not wish to become pregnant but are not using contraception, it does not capture whether these women truly want to be using a form of contraception. Hence, this indicator is predicated on the assumption that all individuals not using a contraceptive method wish to be using one, and therefore have an unmet need (14–16) The indicator, mCPR that captures the number of family planning users in a population, has also been critiqued for capturing women that are married or in union only, as well as for focusing only on those using modern forms of contraception while excluding those using natural methods (15, 16).

The 1994 International Conference on Population Development in Cairo marked a shift for the FP community towards reproductive health and rights, with the international community formally agreeing that “*The aim of family-planning programmes must be to enable couples and individuals to decide freely and responsibly the number and spacing of their children*” (17). Later, in 2012, the London Summit on FP launched FP2020, an international consortium comprised of governments, NGOs and advocates which pledged 2.6 billion USD to provide contraceptive methods to an additional 120 million girls and women in the world's 69 poorest countries by 2020. The 2030 agenda for the Sustainable Development Goals highlighted the need to achieve gender equality as well as universal access to sexual and reproductive health services. As a result, more FP programmes were implemented, utilising demand generation strategies to overcome barriers to non-use of contraception. Although FP programme language now emphasises reproductive rights over demographic targets, they often continue to set numeric goals for contraceptive use, which can enable implicit and subtle forms of coercion, such as restrictions in FP methods provided, limited access for unmarried or adolescent women, or biased counselling—issues that have been documented in the literature (10, 18–21). Furthermore, a power imbalance exists between donor organisations, primarily based in the Global North, which control much of the FP agenda, and the local agencies implementing these programs in the Global South. By directing FP efforts to meet economic goals and numeric targets of increased contraception users instead of prioritising individual rights, these organisations risk perpetuating a form of neocolonial control—wherein Western organisations impose their own values and agendas on communities in the Global South. This dynamic can marginalise local voices in critical decisions that impact their reproductive autonomy and decision-making (22, 23).

The strategy of FP2030 (the updated iteration of FP2020) emphasises equitable, voluntary, rights-based FP and designated three new focus areas: reproductive choice, autonomy and empowerment, and gender equality. The Performance Monitoring and Evidence Working Group (PME WG) within FP2030 states that it applies a human rights and reproductive justice lens. It also advocates for indicators that can capture individual reproductive choice and fertility intention (24). While the move towards prioritising reproductive autonomy has been well-received by the global FP community (25, 26), novel measures of success to capture reproductive and contraceptive autonomy have not yet been introduced. There is a knowledge gap concerning why such indicators have not yet been implemented and how they could and should be adopted.

This paper aims to explore the views of stakeholders in the international FP community- namely workers from funding agencies, governmental institutions, non-governmental organisations (NGOs), as well as academics and reproductive health advocates—on current measures of success of FP programmes. We explore their views on these measures' limitations, ties to coercion as well as barriers to developing and adopting new indicators.

## 2 Methods

### 2.1 Study design and setting

This study is part of a larger multi-layered project to explore the ethical issues within the international family planning community. We conducted a global level study, which is represented in this paper that explores the views of international stakeholders within the FP community on ethical challenges of FP programmes in Francophone West Africa (FWA). We also conducted regional and local level studies that captured the views of implementors of FP programmes in FWA, as well as community members, i.e., men and women living in Senegal on their views of FP programmes in the country. Peer-reviewed papers from the latter two layers will be published separately. In this paper we explore their views on the indicators used to measure the success of FP programmes. We employed a qualitative methodology utilising online semi-structured interviews with 31 participants. The study took place between October 2020 and January 2021, during the COVID-19 pandemic, hence all interviews were conducted online via Zoom. The interviews lasted between 45 min and an hour. All interviews were audio-recorded with the permission of the interviewees. Interviews were conducted in either English or French depending on the preference of the interviewee.

### 2.2 Sampling

We applied a purposive sampling strategy to identify participants working within the field of sexual and reproductive health and FP globally, as well as specifically in Francophone West Africa. We interviewed a total of *n* = 31 participants from five distinct categories of stakeholders: academics (10), advocates (5), government officials (4), NGO personnel (4) and funding agencies' staff (8). Twenty-one of our participants were either global institutions or based in North American and European organisations, while nine were based in West African organisations and one in a sub-Saharan African country. Details of the participants' professional background category, gender and geographic locations are provided in Table 1. At the start of the study, we identified key informants from the FP community, including academics, funding agency workers and advocates. Our key informants provided feedback on our preliminary topic guide to ensure the flow, clarity and relevance of our questions and themes. They also helped us in identifying some of our study participants. We then applied a snowball sampling strategy to identify more participants and continued until saturation (27). Interviewees were contacted via email by the research team to seek consent to participate. Consent forms and information sheets were sent via email prior to each interview.

**Table 1:** Participants' characteristics.

Number	Gender	Profession	Geographic location
P 1	Female	Advocate	North America
P 2	Female	Funder	North America
P 3	Female	Funder	West Africa
P 4	Female	NGO	Europe
P 5	Female	Funder	Europe
P 6	Female	Funder	Global
P 7	Male	NGO	West Africa
P 8	Male	Academic	West Africa
P 9	Female	NGO	Sub-Saharan Africa
P 10	Female	Advocate	Europe
P 11	Male	Advocate	Europe
P 12	Female	Academic	Europe
P 13	Female	Academic	West Africa
P 14	Female	Advocate	North America
P 15	Female	NGO	Europe
P 16	Female	Academic	Europe
P 17	Female	Academic	Europe
P 18	Female	Governmental official	West Africa
P 19	Female	Academic	North America
P 20	Female	Academic	North America
P 21	Female	Academic	North America
P 22	Male	Academic	Europe
P 23	Female	NGO	West Africa
P 24	Female	Governmental official	West Africa
P 25	Female	Governmental official	West Africa
P 26	Female	Governmental official	West Africa
P 27	Female	Funder	North America
P 28	Female	Funder	North America
P 29	Female	Advocate	North America
P 30	Female	Funder	Europe
P 31	Female	Funder	Europe

### 2.3 Data collection methods

We formulated the topic guide based on a scoping literature review, which aimed to understand how FP interventions in sub-Saharan Africa address the ethical challenges raised within the literature (28). Next, we sought feedback from the key informants and adjusted the guide according to their feedback. The questions were open-ended and explored themes related to personal experiences in family planning, links between reproductive health targets and women's empowerment, challenges in FP funding, strategies to enhance women's roles in FP programme design, and solutions to ethical challenges in FP programming.

### 2.4 Data analysis

All interviews were anonymised and transcribed verbatim in the original language, either English or French. French transcripts were translated by French-speaking members of the research team into English to allow all team members to take part in the analysis process. Using NVivo software, we coded the data to identify emerging themes. We applied a thematic analysis approach and utilised deductive analysis first, where we established our codes *a priori* based on the guide themes, after which we applied an inductive analysis, where we generated themes to expand our code book. The coding was done in several stages. First, all team members openly coded on a group of twelve transcripts to allow for the identification of keywords and themes. Next, a code book was developed after data saturation was reached and was used for the first round of coding. The codebook was then refined after team discussion and finalised for the second and final round of coding.

### 2.5 Ethical considerations

All participants were anonymised, and interviews were given unique identification codes. Personal information of individuals and their associated organisations were omitted from transcripts. Participants were aware that collected data may be used in publications and reports after being anonymised and ensuring no deductive disclosure risk. All data is managed in accordance with the Data Protection Act, 1998, and the LSHTM Information Security Policy and Research Data Management Policy. Only members of the research team have access to the anonymised data that is saved on a secured server. Ethical approval was provided on September 1st, 2020 by LSTHM research ethics committee, reference number: 22553/RR/21111.

## 3 Results

The results section presents the views of participants on the measures of success utilised by current FP programmes, including their strengths and limitations, novel indicators to adopt, and the current and anticipated barriers to adopting new measures.

### 3.1 Views on current indicators

Views on measures of success spanned those who believed in complete eradication of current indicators to those that defended them as good indicators that require some adjustments. The majority described the use of mCPR and unmet need as useful in providing a general picture, albeit in an imperfect way, of how an FP programme delivers contraceptive services and the long-term effects of demand generation interventions.

Above all, current measures of success were described as capturing contraceptive uptake while lacking the necessary variables to capture essential markers like autonomy and choice. For example, a funding agency worker was critical of the many programmes, as well as funding agencies, who showcase how programmes help women by only displaying how they have reached specific numerical targets of contraceptive use.

*I feel like women's autonomy is superseded by the desire to get the numbers out. Like “We got this many products out so that must be how many women we've helped!”* P3, Funding agency, Europe.

A few participants vocalised their frustration with the disconnect between what funders promise: a rights-based approach; what they deliver: modern forms of contraception; and how they measure success: through uptake alone.

*FP2020 was, I'll call it reactionary, in that upon being criticized for setting a target that left space open for that* [coercion] *their response was to say “Okay, we'll convene, a group that, promotes the rights-based aspect of it” … .There's actually zero accountability for the rights-based part of it. 120 million additional users. We don't know if there is zero among them who are coerced into using family planning or 120 million of them coerced into using it*. P14, Advocate, North America

Although the previous participant found a positive link between fertility measures and assessing the dependency ratio, others demanded a more thoughtful indicator and criticised simplistic demographic-focused approaches which posit that decreasing the population will lead to a definite improvement of the economy.

*I think it's really problematic when people are like “Oh, if Niger just drops* [TFR] *from twelve to six, they'll achieve the demographic dividend in 2035!” That's not how that works. The demographic dividend's language has become really sexy right now but I think that if you talk to economists, they're like “Wait” … My understanding of the demographic dividend is all other kinds of stuff needs to be in place in order for a country to, achieve the economic benefits of that specific set of events or circumstances.* P29, Advocate, North America.

### 3.2 Room for coercion

A recurrent theme identified by our participants was how current indicators and their related targets allow room for coercion, even if implicit and indirect, and can lead to unintended negative outcomes. A funding agency worker in Europe passionately criticised the insistence on using total fertility rate (TFR) as a measure of success of FP programmes, which she said “*equals so much coercion*” since it can be directly linked to policies that “*impose how many children people should have*”. She believed using this measure directly interferes with the rights of individuals and couples to choose how many children they would like to have. Furthermore, several of our informants described how the focus on measuring contraception uptake within FP programmes may lead to failure of recognising non-use as a valid measure of success even when based on an informed, full and free choice.

Some participants wished to completely eliminate certain terminology rather than redefine or expand on it. Their responses underscore certain terms' links to the historic colonial use of FP programmes to control populations in the Global South.

*I think we just need to banish the word target. Honestly, it's a terrible word in our field that brings up so many terrible past experiences*. P1, Advocate, North America.

Participants from funding agencies reflected on how framing FP programmes' targets around increasing the number of women using modern forms of contraception may incentivise service providers to de-prioritise autonomy and free choice, and impact ethical service delivery. Focusing rigidly on targets was described as applying systemic pressures on providers which can lead to coercive practices within the programmes.

*Occasionally you just see these alarming things like “we're going to go to this community and, within this three-month project we're going to take their mCPR from 6% to 30%”. And you're like “No, that doesn't sound quite right”* Funding agency official, Europe.

*I think one of the foundational challenges that inhibits everyone is that we focus on measuring uptake and not alignment with what the client wants. And so that leaves that room for possible coercion.* P30, Advocate, North America.

*There is an article that came out recently about the systems that can lead to reproductive coercion and how it's not just a relationship with the provider but all the bureaucratic things spinning around and pressure to reach these targets, it can end up being coercive for women experiencing it.* Advocate, North America.

Additionally, participants described that the over-promotion of LARCs in international targets biased the views of service providers towards these methods, meaning they may not offer clients a varied method mix. An advocate describes how, when attending a conference related to FP, she challenged speakers who focus on promoting LARCs:

*They had a statement that they promote LARCs and I said why do you say that, you may think that that's a good thing that you're expanding access to LARCs but somebody can hear that as “I'm supposed to use a LARC”. So I said this is how a lot of this of unintended consequence happens that I think I'm promoting something in a good way, I'm making it available, but somebody else hears, a provider hears, that's the method I'm supposed to be pushing.* P29, Advocate, North America.

Similarly, an advocate described how counting women using *any* form of modern contraception as users is not sufficiently nuanced as it fails to capture if they are using their preferred method of choice.

*Not all uptake, even among people who want family planning, is good uptake. If they want an IUD* [Intrauterine device] *but they're using a condom, they get counted as uptake. When you're like “No, that's actually not a good outcome for them, they're still not using their preferred method!”.* P14, Advocate, North America.

Other participants highlighted how FP programmes overemphasise measuring usage, or uptake of contraception as a measure of success, but not removal of contraception.

*Not long after 2012, there was a big push to make implants available. And one NGO inserted 80,000 implants but they did not provide access to removal, which is much harder. And women had to travel hours to a clinic where there was somebody qualified to remove it if she wanted it removed. That was horrible, it's just unacceptable. And the NGO was saying “Well, you know, it's not going to hurt her”. No! that's not the point. If she wants it removed she should have access to ready removal. Period.* P2, Funding Agency, North America.

Furthermore, mCPR was described as not capturing continuation or discontinuation of use beyond the client-provider interaction.

*It does not capture what happens after leaving the service. “Okay, I'm happy because I offered a method and she went out with the method” but we're really not really understanding what will happen after that woman or girl leaves the door … …  And it happens then that they stop using it or that they don't come back for the follow-up visit. So this shows that someone, had the need but that need was not necessarily satisfied*, P4, iNGO worker, Europe.

### 3.3 Misunderstanding women's needs

Our interviews showcased how participants from different sectors had similar views and frustrations on how the use of indicators like mCPR and TFR made “family planning” synonymous to contraception. One academic critiqued how FP services have become completely focused on promoting limiting and spacing of births while ignoring the needs of those who want to expand their families. A participant criticised the demand satisfied measure and said:

*Family planning in its purest sense is enabling couples to have as many children when they want them as they wish—the infertility or infecundity side of the equation is conspicuously missing. And it is rather hypocritical, I think, that it continues to be totally missing.* P22, Academic, Europe.

Current measures were also described as perpetuating a global standard that all women of reproductive age should be taking a form of contraception, despite many women not wishing to do so.

*You will always have a group of women who do not want to* [use contraception] …* Not all of women need to have family planning and I think it's this idea of the perfect world or global standard for every woman to be using family planning. Honestly, no!* P4, iNGO, Europe.

Furthermore, many participants believed that focusing on measuring unmet need and demand satisfied reflected the naïve outlook of the FP community on fertility desires as a simple dichotomous outcome. In this way, FP programmes categories women into two groups; those who want to get pregnant and those who do not, ignoring how ambivalence is an option of women's fertility desires.

*The problem is that it assumes that women are binary … If I don't want to become pregnant of course I should be using contraception …*  [a researcher] *describes women fertility intentions a continuum … Where on the one hand, there's a woman who would do anything to get pregnant …  She'll do IVF* [*in vitro* Fertilization]*, And on the other end of the spectrum, there's women who would risk their lives to have an abortion, would do anything to not be pregnant. And then there's everybody in the middle. And that changes, day to day, based on their relationship, based on their income, based on, COVID..* *It changes all the time and women do a cost-benefit calculus..That's much more nuanced than you get in.* A15, iNGO-Europe.

### 3.4 Medicalisation of family planning

Family planning services can include non-medical or natural methods, such as fertility awareness-based approaches, and modern methods, such as short acting reversable oral contraceptive pills and LARCs, including implants and intra-uterine devices. Participants described how the medicalisation of FP programmes meant that FP services prioritise modern methods. This was especially apparent to those working on promoting natural methods to FP clients and conducting research in understanding which contraceptive methods women prefer. An advisor from an international funding agency passionately spoke of her frustration with the FP community in belittling natural forms of FP and not providing FP clients with the necessary information and knowledge on how to utilise them properly.

*Within the family planning world I've had a major fight because we have developed these wonderful fertility awareness-based methods like the standard days method and apps based on that. But there's so much inherent prejudice against anything that's knowledge-based within the medicalised family planning field that getting people to understand that this is what women do want, a certain percent of them, and it should be a clear option within our programmes, is still a battle after 25 years of research. So it's always been my biggest frustration that it's still hard to convince people that you need these broader choices for women and men, and that it's very much about helping women to understand their bodies and make their decisions.* P27, Funding agency, North America.

Moreover, the exclusive focus on provision of contraception ends up treating women as clients of contraception rather than whole individuals with different needs related to sexual and reproductive health, which can negate the reproductive justice framework that many international organisations claim to follow.

*It's very difficult to think of contraceptives without connecting it with also maternal and child health, with sexually transmitted infections, with sexual health per se, and in connection with gender and the continuum of violence against women. So, to see family planning as decontextualized from the other things may be so-so. I mean, probably for targets it's easier to market it in that way. But for women and individuals, I think it's problematic.* A16, Academic, Europe.

FP programmes' focus on contraceptive method provision was described as undermining an integrated or holistic approach to improving reproductive rights, for example through social initiatives to empower people to negotiate and access their full rights.

*We often talk about this problem that we have in being able to make the case for a broader human rights approach to sexual reproductive health and not just having that clinical gaze on sexuality and bodily autonomy …* . *I think that a lot of the family planning world sees everything through the prism of a family planning product. And maybe they've slightly forgotten the underlying point of people making choices about family planning. and people get lost along the way there.* P5, Funding agency worker, Europe.

Furthermore, participants recommended expanding FP definitions beyond physical health to take into account factors such as social networks, economics, and religion.

*We cannot approach family planning from just one angle. We need several axes to talk about health issues today. We have made many mistakes in our approaches and we continue to make the same mistakes in our approaches, because we think that health is only health, we forget many other factors. Societal factors are extremely important, religion is very important, the economy is very important, and many things are important.* P6, International funding official, Europe.

### 3.5 Inaccuracies

Several participants highlighted how existing indicators provide inaccurate estimations. For instance, an academic from a West African country told us how measures such as unmet need are not able to capture contraceptive failure:

*When calculating “unmet need” women who are currently using but get pregnant are excluded … . So for us to continue to use that unmet need for family planning, we must find a way of bringing in that contraceptive failure.* P8, Academic, West Africa

Likewise, another academic from a West European country explained some of the issues with calculating indicators like demand satisfied in different contexts.

*The demand satisfied* [indicator]; *the denominator is demand. And demand can be quite small in a country like Niger where desired family size is very high …* *And when demand is low, that means that small increases in contraceptive use shift percent of demand satisfied, by large amounts and people get very excited*. P22, Academic, Europe.

Participants also identified limitations in indicators that aim to measure men's involvement in fertility decision-making resulting from simplistic and culturally non-specific design. An academic provided an example of one organisation that aimed to assess men's involvement in FP use by calculating the number of men accompanying their wives/partners in the clinic. They highlighted the limitations of such measures which fail to capture the cultural and pragmatic challenges that can prevent men from accompanying their wives even though they might be very involved in the FP decision-making.

*Attempts in measuring “gender norms” and men's involvement in the decision-making process, is it accurate? Representative? Useful? Naively looking at men who accompany women to the clinic* [is not helpful]*, what about places where this is not culturally appropriate or simply not feasible since why would the man take day off to accompany his partner to a “regular” checkup?* P12, Academic, Europe

### 3.6 Funding pressures

Throughout our interviews we asked participants to reflect on possible tensions they face when receiving or providing funding for FP programmes. Many participants described how funding agencies have heavily influenced the narrative of global FP through setting numerical targets, as highlighted above. Participants were clear that organisations delivering FP programmes felt pressure to meet those targets to not lose out on future funding.

*The pressure was also on NGOs to reach the targets set by funders, “when donors establish a certain goal and then you have to reach it otherwise you will not be funded for the next year or …”* P4, iNGO, Europe.

*You know that very well, when they give funding it is to get results. That's absolutely, that's the rule. It's based on indicators.* P24, Governmental official, West Africa.

Participants voiced frustration at the short timeframes in which funders expected change and their lack of understanding of the time needed to generate behaviour change.

*Funders when they have short projects and want to measure immediate “significant differences”* P21, Academic, North America.

* … they go “Okay, find me a country where we can get a quick hit”. And it's like “Behaviour change is not a quick hit!”. and* [Funder] *comes in going “I sold this many computers in this many years, and this is what I expect to do with this”.* P3, International Funding agency worker

Nonetheless, participants acknowledged the complex pressures and challenges faced by funders. For instance, several participants highlighted that governmental funding agencies operate under intense scrutiny. These agencies often strive to avoid any negative outcomes from their programmes becoming public, driven by concerns about accusations of unethical practices, which could potentially lead to funding cuts or suspension of FP programmes

*They* [funders] *have some significant challenges in trying to measure the right things that are probably based in a bit of fear and reluctance to reveal potential negative incidents that take place under their funding … I think the concerns about shifting towards a measurement model that measures alignment with someone's reproductive intentions rather than just uptake is that it will start to reveal more negative things than what we currently measure. If we start measuring coercion we will start seeing coercion. And “allies” within that funding institution are reluctant to move towards a more ethical and better model because they know that the political backlash could be incredibly problematic towards the funding portfolio, which is still obviously the largest source of family planning aid in the world. So that's incredibly complicated*. P14, Advocate, North America.

### 3.7 Adoption of new measures

Although participants expressed a desire for programmes to incorporate measures that can capture choice and autonomy to provide a more “*comprehensive picture*,” many of those measures were described as “*hard since they don't lend themselves to numbers, they're indicators that measure norms and power*.” Advocate, Europe.

Difficulties also related to the difficulty in defining free choice and agency, which rarely operate in isolation of the norms, values and power dynamics in an individual's social context.

[Agency] *might mean different things in different settings because of different ways of understanding family planning or ways of understanding family … So even within contexts where you think organisations impose something, people have agency to decide that they don't want certain things. Or they use all sorts of mechanisms to take those programming, for example, and make them work for themselves within the context that they live in*. Advocate, Europe.

*It's possible that the partners are influencing those choices, but you want to try to ensure that they are doing it for themselves on the partner's advice. I mean, You don't really take reproductive choices alone if you're in a relationship, usually, whoever you are.* P10, Funding agency official, Europe.

Above all, participants emphasised the need for women themselves, particularly those in the Global South, to be involved in decision-making process. As one funding agency worker put it “*We're still operating in this sort of colonial mindset of ‘Here we are making the decisions for these group of people*’”. They underlined how FP programme design is often shaped by decision-makers based in the Global North and by predominantly male-led spaces. Others challenged the latter perception, highlighting that the FP community is largely led by older, white, western women who bring a second-wave feminist approach that may lack a sufficiently intersectional lens. The following participant further explains:

“*Even the rights-based part of it comes from an antiquated traditional approach that I find wholly insufficient. It is literally mostly American White women in their sixties who define what that rights-based approach should look like. And that just blows my mind. I'm like ‘This is, not only insufficient for what it is but we don't even have the right people calling that out’. I think they have one or two people now who they're starting to bring in who are younger, from the Global South, who are taking a lens of intersectionality, as radical as that might seem for this community. And I think it's really exciting what they're doing but that hasn't really translated into their rights-based family planning push, which is tough to watch and to be a part of.”* P14, Advocate, North America.

Participants underlined that women's representation and participation in decision-making around targets and funding is essential to rebalance power dynamics in FP programme design. However, challenges in identifying who can and should adequately represent women were also mentioned.

*I think that the biggest issue then is who speaks for women. And who speaks. and whose voices are heard. And then one of the questions becomes at what point is that voice representative? But then there's an important question with that, which is what level of knowledge is needed from people who speak for other people. And there's a certain tension there because I also see that people are more listened to, they're more heard by government officials if they have more credibility. And I also recognize that there's a co-optation that comes with that process and that that tension is important.* P19, Academic, North America.

When asked what the ideal indicators would look like, participants emphasised the importance of going beyond the quantitative indicators that are easy to measure as boxes to tick off. Rather than re-inventing the wheel, several suggested adopting measures from other disciplines like measures from the psychosocial literature, like self-efficacy and community support. The main variables that almost all participants identified as the priority to measure were autonomy and choice. Other participants described the need for indicators to capture other dimensions of contraceptive use like “*continuation, ability to switch, communication with partner*” as well as “*self-efficacy and community support*” -Funding Official, North America. Other suggestions for novel measures focused on capturing male involvement in FP decision-making as well as “*number of women currently unhappy with their contraceptive method or using it inconsistently*”. P2, Advocate North America.

Many highlighted the importance of having a mix of indicators to capture a balanced picture of programme implementation and triangulate perspectives.

*I don't think it can ever be one indicator. I think that there has to be a mix of quantitative, qualitative and policy indicators. Understanding women's perspectives, as well as providers, as well as the community in general. Triangulation of those three.* P19, Academic, North America.

Several participants suggested integrated or holistic approaches to improving reproductive rights by integrating medical solutions with social ones to help empower women in negotiating what they think is best for them. Hence, FP programmes needed to develop or adopt measures of success that can capture nuanced variables such as reproductive rights and wellbeing. In this regard, one funding official said:

*I would model it on the reproductive justice movement in the US, which is much more focused on a much broader way of thinking about reproductive choices. Not just about contraceptive choice but about having the right to raise your children in a safe environment, have the number of children you want and so on. So reproductive wellbeing would be my goal*. P2, Funding official, North America.

## 4 Discussion

Our study examines FP stakeholders' views on current indicators, revealing concerns that a focus on contraception uptake over autonomy fosters implicit coercive practices. Our participants described inaccuracies and limitations of current indicators, which is echoed in the wider literature, where unmet need, demand satisfied and fertility measures like TFR have been described as failing to capture women's true reproductive desires as well as the voluntariness of contraceptive use (14, 29–30).

Several studies highlight implicit coercive practices in FP programmes, including over-promotion of LARCs, biased counselling for post-partum women, injectables being used without women's consent and providers refusing to remove methods upon request (20, 31–35). Our participants described how implicit coercion is driven by pressures on providers to meet quantitative targets at the expense of quality care, with non-use or removal of contraception often measured as a failure, which has been reported by several research papers (9, 31, 36, 37). Despite attempts to develop autonomy-based indicators, their adoption remains limited, underscoring an urgent need for improved FP measures that prioritize autonomy and agency.

Additionally, the narrow definition of FP as contraceptive provision disregards those seeking fertility support, while assuming all women without childbearing intentions desire using modern contraception. Prioritisation of modern methods, and the assumption that all women of reproductive age wish to be using a method, overlooks individuals with a preference to use natural methods or to opt out of any contraception use. Similarly, the literature has reported how focusing exclusively on measuring modern contraceptive methods may lead to under-reporting of use of natural methods and may disregard women's preference in choosing their ideal contraceptive method (16, 29).

Our results hinted at how the assumption that population reduction may directly lead to economic development is a too simplistic view. As Senderowicz and Valley (38) argue, family planning alone cannot drive sustainable development, which requires addressing multiple factors like healthcare, education, infrastructure, and income inequality. A narrow focus on population control risks neglecting these essential elements, which are crucial for equitable progress (38). Hence, adopting a reproductive justice framework was described as an essential step in improving FP services. Likewise, our results highlighted the need for indicators to reflect diverse reproductive goals and relational agency, acknowledging influences from partners and social networks. There is a growing support for the adoption of an intersectional reproductive justice framework which would situate FP in a more holistic framing in relation to its wider role in shaping women's lives and autonomy (39, 40).

### 4.1 Strengths and limitations

One strength of our study was the range of participants, who came from different geographic locations and professional backgrounds. Although the majority of the participants were from the Global North, this was mostly because most of the funding agencies were based within that region. Although a limitation of this study may be not gaining the perspective of FP service providers, we conducted interviews with this group within other layers of our project and will report those findings in project reports. It is important to highlight that our interviews were conducted between October 2020 and January 2021 before the launch of the FP2030 strategy. Since then, the language of the FP2020/FP2030 partnership evolved, and we encourage future research to assess if and how FP programmes and stakeholders' perspectives on them may have changed as a result.

### 4.2 Implications

Our results suggest that FP programmes continue to be used as vehicles for shaping demographic trends in the Global South by actors in the Global North, despite this having been ostensibly rejected by the international community in 1994. The perspectives of our participants reflect findings from another of our studies, which highlights the ongoing and explicit use of population control narratives by international FP funders (9).

Our results further highlight power imbalances in the international development community, in which decisions are usually made by Global North actors on behalf of individuals living in the Global South. FP programmes continue to be built on a Euro-American model of reproductive and contraceptive desires which may disregard the rich, diverse historical approaches to reproductive desires and contraceptive practices across different communities and religious traditions. Although family planning as a concept is not a Western invention, modern forms of contraception, as well as linking economic growth and environmental sustainability to family planning may often reflect Western-centric perspectives. We, hence, argue for the need to decolonise not just the provision of FP but the narrative as well. We further argue to ensure women's voices from both the Global North and South are included in the decision-making processes within the FP community.

Change is needed to meet the goal of contraceptive and reproductive autonomy for clients of FP services. The FP community must engage in efforts to translate emerging evidence on the nuances of fertility desires, reproductive rights and decision-making processes in different contexts into programme design. Better inclusion of women in decision-making processes will enable programmes to be designed in a way that fulfils a panoply of fertility desires, including contraceptive non-use. Furthermore, applying an intersectional lens to research as well as to policymaking, especially including the voices of FP users and non-users from the Global South, is essential to ensure better representation of women's needs and voices.

We urge funding agencies to invest in developing and implementing measures of FP programme success that move away from prioritising increasing the number of users alone, and towards capturing autonomous, full, informed and free choice to use or not use contraception.

## 5 Conclusion

Our study emphasises the need for FP programmes to move beyond measuring success through quantitative indicators focused on modern contraceptive uptake alone to prioritise women's reproductive and contraceptive autonomy and choice, as well as their inclusion in policy and programme design and decision-making. Current metrics, focused predominantly on contraceptive uptake, often fail to capture the full spectrum of reproductive desires and choices, including those of individuals who seek to expand their families, and can reinforce implicit coercive practices. There are socio-political complexities and ethical dilemmas embedded within FP practices, particularly related to coercive practices and power imbalances between the Global North and South, which must be adequately and explicitly addressed in FP programme design. Moving forward, we call for the decolonisation of FP programme design and recommend policymakers and programme designers apply intersectional approaches that prioritises reproductive justice, with funding agencies supporting metrics that ensure informed and voluntary choice, rather than simply increasing the number of users or promising economic prosperity. Expanding FP services to provide fertility treatments as well as contraception can better meet the diverse reproductive needs of individuals globally, ensuring that FP programs truly prioritise and support reproductive autonomy rather than dictate what it should look like.

## Data Availability

The datasets presented in this article are not readily available in order to protect the anonymity of the participants due to risk of deductive disclosure. Requests to access the datasets should be directed to nour.horanieh@lshtm.ac.uk.

## References

[1] Cleland J Bernstein S Ezeh A Faundes A Glasier A Innis J. Family planning: the unfinished agenda. Lancet. (2006) 368(9549):1810–27. 10.1016/S0140-6736(06)69480-4 17113431

[2] Coale AJ Hoover EM. Population Growth and Economic Development in Low-Income Countries. Princeton, NJ: Princeton University Press (1958). p. 6–25.

[3] Frey M. Neo-Malthusianism and development: shifting interpretations of a contested paradigm. J Glob Hist. (2011) 6(1):75–97. 10.1017/S1740022811000052

[4] Hodgson D Watkins SC. Feminists and neo-malthusians: past and present alliances. Popul Dev Rev. (1997) 23(3):469–523. 10.2307/2137570

[5] Bracke MA. Women’s rights, family planning, and population control: the emergence of reproductive rights in the United Nations (1960s–70s). Int Hist Rev. (2022) 44(4):751–71. 10.1080/07075332.2021.1985585

[6] Pollack Petchesky R. From population control to reproductive rights: feminist fault lines. Reprod Health Matters. (1995) 3(6):152–61. 10.1016/0968-8080(95)90172-8

[7] Nandagiri R. What’s so troubling about “voluntary” family planning anyway? A feminist perspective. Popul Stud. (2021) 75(supp1):221–34. 10.1080/00324728.2021.1996623 34902284

[8] Ewig C. Hijacking global feminism: feminists, the catholic church, and the family planning debacle in Peru. In: Elliott CM, editor. Global Empowerment of Women. New York, NY: Routledge (2007). p. 327–49.

[9] Witt A Montt-Maray E Fall M Larson E Horanieh N. Putting our money where our mouth is? The degree of women-centred family planning in the era of FP2020. Front Glob Womens Health. (2023) 4:1148851. 10.3389/fgwh.2023.1148851 37325793 PMC10262847

[10] Cleland J Harbison S Shah IH. Unmet need for contraception: issues and challenges. Stud Fam Plann. (2014) 45(2):105–22. 10.1111/j.1728-4465.2014.00380.x 24931071

[11] Seltzer JR. The Origins and Evolution of Family Planning Programs in Developing Countries—University of Edinburgh. Santa Monica, CA: Rand (2002). Available at https://discovered.ed.ac.uk/discovery/fulldisplay?vid=44UOE_INST:44UOE_VU2&tab=Everything&docid=alma9924040252502466&lang=en&context=L&query=sub,exact,%20Clinical%20trials (Accessed November 25, 2024).

[12] Bradley SEK Casterline JB. Understanding unmet need: history, theory, and measurement. Stud Fam Plann. (2014) 45(2):123–50. 10.1111/j.1728-4465.2014.00381.x 24931072 PMC4369378

[13] Belaid L Dumont A Chaillet N Zertal A De Brouwere V Hounton S Effectiveness of demand generation interventions on use of modern contraceptives in low- and middle-income countries. Trop Med Int Health. (2016) 21(10):1240–54. 10.1111/tmi.12758 27465589

[14] Fabic MS. What do we demand? Responding to the call for precision and definitional agreement in family planning’s “demand” and “need” jargon. Glob Health Sci Pract. (2022) 10(1):e2200030. 10.9745/GHSP-D-22-00030 35294394 PMC8885353

[15] Cahill N Sonneveldt E Stover J Weinberger M Williamson J Wei C Modern contraceptive use, unmet need, and demand satisfied among women of reproductive age who are married or in a union in the focus countries of the Family planning 2020 initiative: a systematic analysis using the Family planning estimation tool. Lancet. (2018) 391(10123):870–82. 10.1016/S0140-6736(17)33104-5 29217374 PMC5854461

[16] Rossier C Senderowicz L Soura A. Do natural methods count? Underreporting of natural contraception in urban Burkina Faso. Stud Fam Plann. (2014) 45(2):171–82. 10.1111/j.1728-4465.2014.00383.x 24931074

[17] UNFPA. United nations population fund. Programme of action. Adopted at the International Conference on Population and Development, 5–13 September 1994, Cairo (2014). Available at https://www.unfpa.org/sites/default/files/pub-pdf/programme_of_action_Web%20ENGLISH.pdf (Accessed 28, November, 2022).

[18] Hendrixson A. Population control in the troubled present: the “120 by 20” target and implant access program. Wiley Online Libr. (2018) 50(3):786–804. 10.1111/dech.12423

[19] RamaRao S Jain AK. Aligning goals, intents, and performance indicators in family planning service delivery. Stud Fam Plann. (2015) 46(1):97–104. 10.1111/j.1728-4465.2015.00017.x 25753061

[20] Senderowicz L. I was obligated to accept”: a qualitative exploration of contraceptive coercion. Soc Sci Med. (2019) 239:112531. 10.1016/j.socscimed.2019.112531 31513932

[21] Bendix D Foley E Hendrixson A Schultz S. Targets and technologies: sayana press and Jadelle in contemporary population policies. Gend Place Cult. (2020) 27(3):351–69. 10.1080/0966369X.2018.1555145

[22] Stevens-Uninsky M Barkhad A MacDonald T Perez A Mbuagbaw L. Decolonization in sexual and reproductive health research methods: protocol for a scoping review. JMIR Res Protoc. (2023) 12(1):e45771. 10.2196/45771 37058333 PMC10148217

[23] Merchant EK. American demographers and global population policy in the postwar world. Mod Am Hist. (2021) 4(3):239–61. 10.1017/mah.2021.22

[24] FP2030. Family Planning 2030. (2024) FP 2030 PWE Working Group. Available at: https://www.fp2030.org/about/pme-working-group/ (Accessed August 21, 2024).

[25] Kamuyango A Arora SK Raney L Ali AK Chandra-Mouli V. FP2020 And FP2030 country commitments: a mixed method study of adolescent and youth sexual and reproductive health components. Glob Health Sci Pract. (2024) 12(5):e2400223. 10.9745/GHSP-D-24-00223 39433424 PMC11521563

[26] FP2030. The FP2030 Strategy—Family Planning 2030. (2024). Available at: https://www.fp2030.org/page/fp2030-strategy-document/ (Accessed 21 August, 2024).

[27] Naderifar M Goli H Ghaljaie F. Snowball sampling: a purposeful method of sampling in qualitative research. Strides Dev Med Educ. (2017) 14:e67670. 10.5812/sdme.67670

[28] Montt-Maray E Adamjee L Horanieh N Witt A González-Capella T Zinke-Allmang A Understanding ethical challenges of family planning interventions in sub–Saharan Africa: a scoping review. Front Glob Womens Health. (2023) 4:1149632. 10.3389/fgwh.2023.1149632 37674903 PMC10478786

[29] Senderowicz L Maloney N. supply-side versus demand-side unmet need: implications for family planning programs. Popul Dev Rev. (2022) 48(3):689–722. 10.1111/padr.12478 36578790 PMC9793870

[30] Speizer IS Corroon M Calhoun L Lance P Montana L Nanda P Demand generation activities and modern contraceptive use in urban areas of four countries: a longitudinal evaluation. Glob Health Sci Pract. (2014) 2(4):410–26. 10.9745/GHSP-D-14-00109 25611476 PMC4307858

[31] Senderowicz L. Contraceptive autonomy: conceptions and measurement of a novel family planning indicator. Stud Fam Plann. (2020) 51(2):161–76. 10.1111/sifp.12114 32358789

[32] Senderowicz L Pearson E Hackett K Huber-Krum S Francis JM Ulenga N I haven’t heard much about other methods’: quality of care and person-centredness in a programme to promote the postpartum intrauterine device in Tanzania. BMJ Glob Health. (2021) 6(6):e005775. 10.1136/bmjgh-2021-005775 34162627 PMC8230964

[33] Towriss CA Phillips TK Brittain K Zerbe A Abrams EJ Myer L. The injection or the injection? Restricted contraceptive choices among women living with HIV. Sex Reprod Health Matters. (2019) 27(1):1628593. 10.1080/26410397.2019.1628593 31533578 PMC6754114

[34] Britton LE Williams CR Onyango D Wambua D Tumlinson K. When it comes to time of removal, nothing is straightforward”: a qualitative study of experiences with barriers to removal of long-acting reversible contraception in Western Kenya. Contracept X. (2021) 3:100063. 10.1016/j.conx.2021.100063 33912827 PMC8063731

[35] Yirgu R Wood SN Karp C Tsui A Moreau C. You better use the safer one… leave this one”: the role of health providers in women’s pursuit of their preferred family planning methods. BMC Womens Health. (2020) 20(1):170. 10.1186/s12905-020-01034-1 32787924 PMC7425019

[36] Hardee K Harris S Rodriguez M Kumar J Bakamjian L Newman K Achieving the goal of the London summit on family planning by adhering to voluntary, rights-based family planning: what can we learn from past experiences with coercion? Int Perspect Sex Reprod Health. (2014) 40(4):206–14. 10.1363/4020614 25565348

[37] Cole MS Boydell V Hardee K Bellows B. The extent to which performance-based financing programs’ operations manuals reflect rights-based principles: implications for family planning services. Glob Health Sci Pract. (2019) 7(2):329. 10.9745/GHSP-D-19-00007 31249026 PMC6641818

[38] Senderowicz L Valley T. Fertility has been framed: why family planning is not a silver bullet for sustainable development. Stud Comp Int Dev. (2023) :1–32. 10.1007/s12116-023-09410-2 PMC12332515 40785973

[39] Cadena DS Chaudhri A Scott C. Contraceptive care using reproductive justice principles: beyond access. Am J Public Health. (2022) 112(S5):S494–9. 10.2105/AJPH.2022.306915 35767782 PMC10461485

[40] Gilliam ML Neustadt A Gordon R. A call to incorporate a reproductive justice agenda into reproductive health clinical practice and policy. Contraception. (2009) 79(4):243–6. 10.1016/j.contraception.2008.12.004 19272493

